# Study on the Factors Influencing the Adsorption Mechanism of CSH Gel for Chloride Ions

**DOI:** 10.3390/ma17225464

**Published:** 2024-11-08

**Authors:** Shijie Liu, Suping Cui, Hongxia Guo, Yali Wang, Yan Zheng

**Affiliations:** 1 College of Mechanical Engineering, North China University of Science and Technology, Tangshan 063210, China; 2 College of Materials Science and Engineering, Beijing University of Technology, Beijing 100124, China

**Keywords:** calcium silicate hydrate gel, adsorption properties, chloride ions, adsorption mechanism

## Abstract

Calcium silicate hydrate (CSH) gel is an important hydration product of cement, significantly influencing the coagulation and hardening processes, as well as the mechanical properties, volume stability, and durability of cement. Moreover, it plays a crucial role in the adsorption of harmful ions. In this study, CSH gel was synthesized through the precipitation of calcium acetate and sodium silicate and was subsequently used to adsorb chloride ions. The results indicated that when the calcium-to-silicon ratio was 1.2, the CSH gel exhibited excellent adsorption performance for chloride ions introduced via CaCl_2_ and NaCl, with adsorption capacities of 17.45 mg·g^−1^ and 8.06 mg·g^−1^, respectively. The adsorption of chloride ions in CSH gel primarily occurs due to the physical adsorption of chloride ions on the surface and within the internal pores of the CSH gel, accompanied by a displacement reaction between hydroxide ion and chloride ions.

## 1. Introduction

Chloride ion wastewater is one of the most common types of wastewaters generated in industries such as printing, dyeing, petroleum, and chemicals [1,2,3]. If this wastewater is not adequately treated before being discharged into the natural environment, it can lead to soil salinization and disrupt the ecological balance [4,5]. The concentration of chloride ions in surface water and groundwater in coastal and saline–alkali areas is notably high, and direct use of this water can negatively impact industrial production. Additionally, elevated chloride ion levels can compromise the accuracy of scientific experiments. Therefore, it is essential to remove chloride ions from water efficiently, rapidly, and cost-effectively [6,7,8,9,10]. Currently, commonly used methods for the removal of chloride ions include precipitation, adsorption, separation, and oxidation [11,12,13,14].

The adsorption process of CSH for chloride ions is complex, and numerous scholars have conducted relevant studies on this topic. Tang et al. [15] found that the chloride ions binding capacity of concrete is largely dependent on the content of the CSH gel within the concrete. They established a relationship between bound chloride ions and free chloride ions in concrete, which can be described using the Freundlich isothermal adsorption model. Zibara et al. [16] discovered that the chloride ions binding capacity of CSH increases with a higher calcium-to-silicon ratio (C/S). Zhou et al. [17] systematically studied the effects of the C/S ratio on the adsorption behavior of CSH through experimental tests and molecular dynamics simulations. Their findings indicated that calcium ions play a crucial role in determining the surface potential in the system and significantly affect chloride ions adsorption. In their molecular dynamics simulations, they observed that an increase in the C/S ratio leads to the breaking of long chains into shorter, defective chains, with the potential for these shorter chains to bond with more calcium ion. High calcium concentrations were found to facilitate the adsorption of chloride ions, resulting in CSH with a higher C/S ratio exhibiting better chloride ions adsorption capacity.

Due to the complex composition of cement-based materials, some researchers have synthesized CSH to observe its chloride ions binding properties in simulated solutions. Gomi et al. [18] analyzed the chloride ion binding capacity of synthesized CSH and found that the chloride ions adsorption capacity is related to its structure. They determined that when the average chain length of CSH is around four, the adsorption of chloride ions is maximized. Furthermore, calcium ions can promote an increase in the chain length of CSH, thereby enhancing its chloride ions binding ability. Hiroshi et al. [19] mixed their prepared CSH with a NaCl solution and found that the binding of chloride ions to CSH adhered to the Langmuir isothermal adsorption model, with a maximum chloride ions adsorption capacity of 0.6 mmol·g^−1^.

## 2. Materials and Equipment

Calcium acetate, water glass (mass fraction is 40%), sodium chloride (NaCl), and calcium chloride (CaCl_2_) were all the reagents used, and they are analytical grade.

Experimental and testing equipment mainly included an X-ray diffractometer (XRD-7000, Kyoto, Japan), specific surface area analyzer (TriStar II 3020, for Kyoto, Japan), scanning electron microscope (Hitachi S-3400N, Tokyo, Japan), inductively coupled plasma emission spectrometer (Optima 7000DV, Waltham, MA, USA), and 400M Solid-State Nuclear Magnetic Resonance Spectrometer (Avance neo 400M, Allston, MA, USA).

## 3. Results

### 3.1. Preparation of CSH Gel

**Figure 1:**
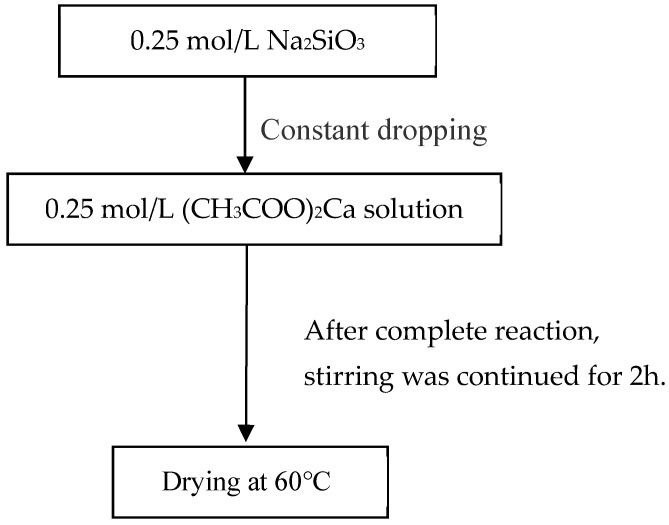
Preparation of CSH gel.

Using calcium acetate as the calcium source and sodium silicate (40% by mass) as the silicon source, CSH gel was prepared using a precipitation method. The calcium-to-silicon ratio was set at 1.2, the pH of the reaction system was 11.0, and the reaction temperature was 30 °C for the preparation of the CSH gel (Figure 1).

Firstly, the phase composition, chemical structure, and surface morphology of the CSH gel were analyzed. The phase composition of the CSH gel was determined using X-ray diffraction analysis. The surface morphology of the CSH gel was examined using a high-resolution scanning electron microscope. Additionally, the structure of the silica tetrahedra in the CSH gel was investigated using a 400 MHz solid-state nuclear magnetic resonance spectrometer.

#### 3.1.1. Phase Composition

**Figure 2:**
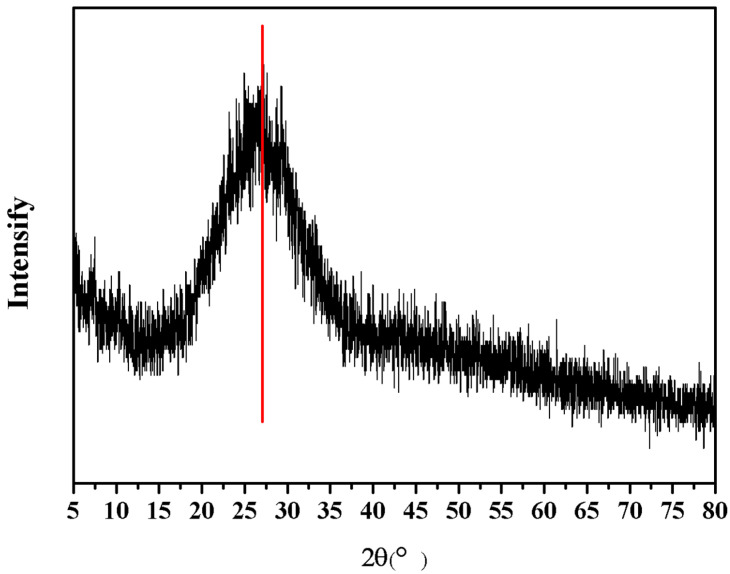
XRD pattern of CSH gel.

XRD analysis of the CSH gel was conducted, and the resulting spectrum is presented in Figure 2. From the figure, it is evident that there is a distinct amorphous “steamed bread” peak in the range of 2θ around 27 degrees, indicating that the prepared CSH gel was entirely amorphous. The gel structure of amorphous CSH contains silicon-oxygen tetrahedra [SiO_4_]^4−^, which include non-bridging oxygen structures that are considered potential sites for chloride ions adsorption.

**Figure 3:**
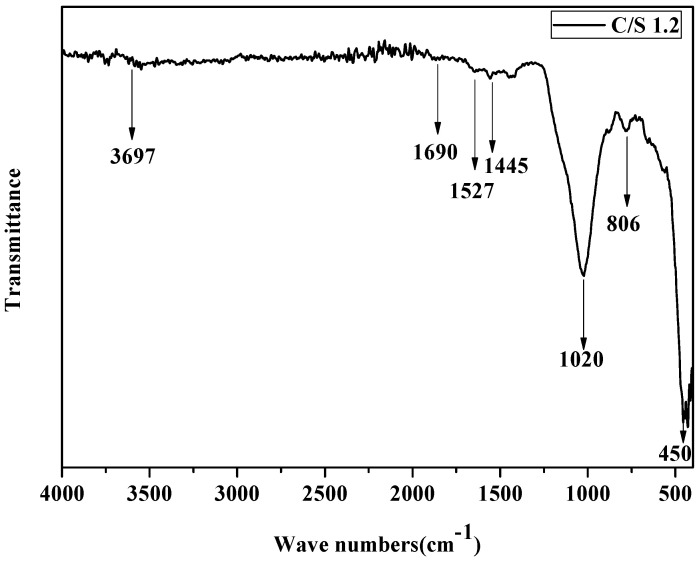
Infrared spectrum of CSH gel.

Due to the varying positions of the [SiO_4_]^4−^ tetrahedra, each chemical bond in the CSH gel exhibits different characteristic vibrational wave numbers. In contrast to XRD, Fourier transform infrared (FTIR) spectroscopy can reveal the structure of substances at group and atomic levels. Therefore, in this paper, in addition to XRD and SEM analyses, the structure of the samples was examined in detail using Fourier infrared spectroscopy. The prepared samples were analyzed using a Fourier transform infrared spectrometer, and the results are presented in Figure 3.

In the infrared spectrum, CSH contains O-Si-O, Si-O-Si, Si-O(Q^1^), Si-O(Q^2^), O-Ca-O, H_2_O, -OH, and other groups, and its peak positions are at 450, 650, 816, 970, 1445, 1640 and 330, respectively. Figure 3 is the Fourier infrared spectrum of synthetic CSH. As can be seen from the figure, the prepared C-S-H sample showed a -OH vibration peak at 3697 cm^−1^, H_2_O vibration peak at 1690 cm^−1^, O-Ca-O vibration peak at 1445 cm^−1^, and Si-O(Q^2^) vibration peak at 1020 cm^−1^. The vibration peak of Si-O(Q^1^) is displayed at 806 cm^−1^, and the vibration peak of O-Si-O is displayed at 450 cm^−1^, which corresponds to the wave number range of each group of CSH, which indicates that the prepared sample had a relatively pure CSH structure.

#### 3.1.2. Structural Composition

The content of Q^n^ structural units in the CSH gel can be determined using the ^29^Si spectrum obtained from solid-state NMR measurements, which allow for the calculation of the network polymerization degree of the CSH gel. It is generally accepted that a lower degree of network polymerization in the CSH gel structure correlates with higher activity. The polymerization degree of the silicate polyhedra was analyzed through NMR, primarily by examining the shifts in the NMR peaks to assess changes in the polymerization degree of the CSH gel.

Based on the number of bridging oxygen atoms coordinated around silicon (Si), the structures can be classified into five categories: Q^0^, ^Q1^, Q^2^, Q^3^, and Q^4,^ where n in Qn represents the number of bridging oxygen atoms surrounding Si. In the CSH gel structure, the breaking of each Si-O-Si bond alters the coordination structure of Si with respect to the bridging oxygen. Generally, the value of the relative bridging oxygen number (RBO) is negatively correlated with the activity of the CSH gel. A higher relative bridging oxygen number (RBO) indicates a greater degree of polymerization in the network system, which in turn results in lower activity [20].

**Figure 4:**
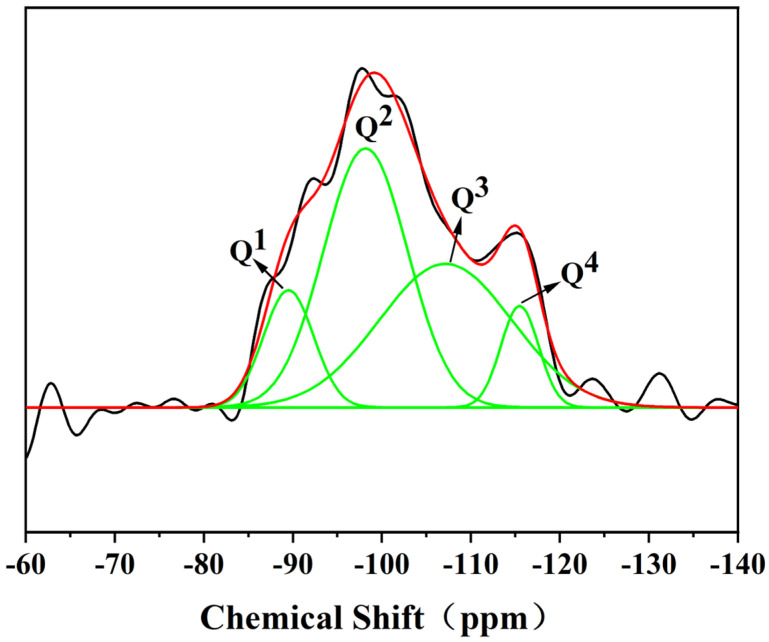
^29^Si NMR spectra deconvolution of CSH gel (The black line was measured, and the red line is the fitted curve).

To determine the content of different silicon structural units in the CSH gel and calculate the corresponding network polymerization degree, Origin (2018, 64-bit version) software was used to deconvolute the ^29^Si spectrum of the CSH gel. The processing was performed using a Gaussian function curve fitting method. After deconvolution, the relative area of each resonance peak could be obtained. The peak area in the NMR data represents the relative content of [SiO4]^4−^ silicon-oxygen tetrahedra in various polymerization states. Figure 4 illustrates the schematic diagram of Gaussian curve fitting for the ^29^Si spectrum of the CSH gel.

As shown in the figure, the chemical shift in the siloxane tetrahedron in the CSH gel structure primarily ranged from −80 to −120 ppm. The polymerization states of the siloxane tetrahedra in the CSH gel included the dimerization state or chain terminal group represented by Q^1^ type, the chain intermediate group represented by Q^2^ type, the siloxane tetrahedron represented by Q^3^ type with a double-chain polymerization structure or layered structure, and the siloxane tetrahedron with a Q^4^ type three-dimensional network structure. The peak shapes of the four types of polymerized silicon-oxygen tetrahedra were relatively broad, indicating that the prepared CSH gel was amorphous.

**Figure 5:**
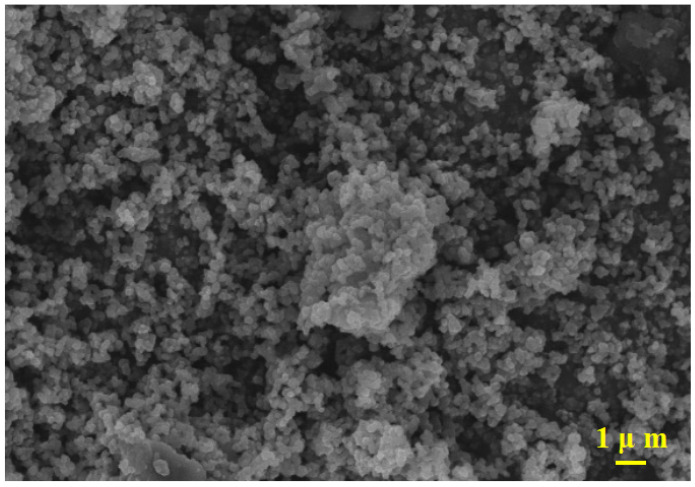
Surface morphology of the prepared CSH gel.

The surface morphology of the CSH gel was analyzed using a scanning electron microscope (SEM), and the results are presented in Figure 5. Due to the rapid reaction between calcium acetate and sodium silicate, crystal nuclei could not form, resulting in the formation of CSH gel with a highly active surface in the absence of a growth environment. As shown in the figure, the primary morphology of the prepared CSH gel was spherical, with distinct particles and an uneven surface. The CSH gel exhibited a very rough surface structure and a large specific surface area, making it advantageous for use as an adsorbent to capture chloride ions.

### 3.2. Effect of Chloride Ions Source on the Adsorption Capacity of CSH Gels for Chloride Ions (Chloride Ions)

**Figure 6:**
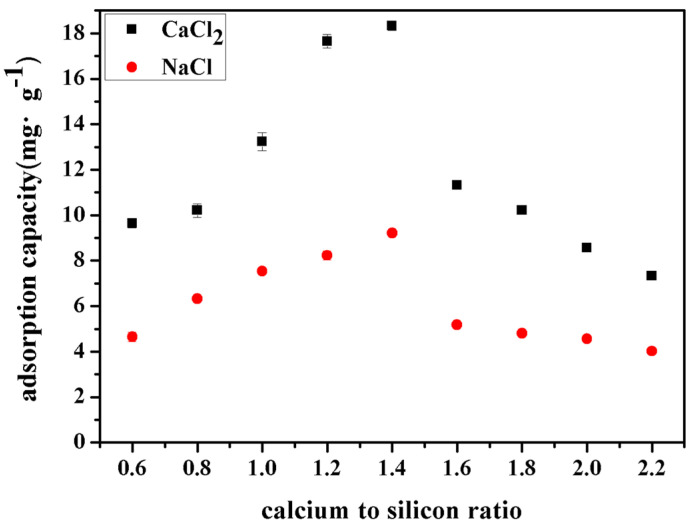
Adsorption capacity of CSH gel for chloride ions from different sources.

This study compares the adsorption capacity of CSH gels for chloride ions derived from different sources, as illustrated in Figure 6. Chloride ions were introduced in the form of calcium chloride ions (CaCl_2_) and sodium chloride ions (NaCl), each at a concentration of 3.0 mol·L^−1^ of chloride ions. It can be observed that as the calcium–silicon ratio increased, the CSH gel exhibited a higher adsorption capacity for chloride ions from CaCl_2_, while showing a slightly lower adsorption capacity for chloride ions introduced via NaCl. Notably, when the calcium–silicon ratio was 1.2, the CSH gel demonstrated optimal adsorption performance for chloride ions from both CaCl_2_ and NaCl, with adsorption capacities of 17.45 mg·g^−1^ and 8.06 mg·g^−1^, respectively. This enhancement is attributed to the ability of calcium ions to promote an increase in the chain length of CSH gels, which facilitates the adsorption of chloride ions by CSH gels [18].

### 3.3. Mechanism Analysis of Chloride Ions Adsorption by CSH Gel

According to the research literature, there are two primary mechanisms for the adsorption of chloride ions in CSH gel: physical adsorption and chemical adsorption. Chemical adsorption occurs due to a chemical reaction between the CSH gel and the chloride ions, making this mode of adsorption more stable. The physical adsorption of chloride ions in CSH gel can primarily be explained by the Electric Double Layer Theory. This theory posits that the electric double layer consists of a stationary layer of charge attached to the surface of the adsorbent, along with a mobile diffusion layer. The potential difference between these two layers determines the adsorption capacity of the adsorbent for chloride ions [21,22,23,24].

To further investigate the mechanism of chloride ions adsorption in CSH gel, we characterized the composition, structure, and morphology of the CSH gel following chloride ions adsorption using X-ray diffraction (XRD), Fourier transform infrared spectroscopy (FTIR), solid-state nuclear magnetic resonance (SNMR), scanning electron microscopy (SEM), energy-dispersive spectroscopy (EDS), and transmission electron microscopy (TEM).

#### 3.3.1. Changes in Composition and Chemical Bonds

**Figure 7:**
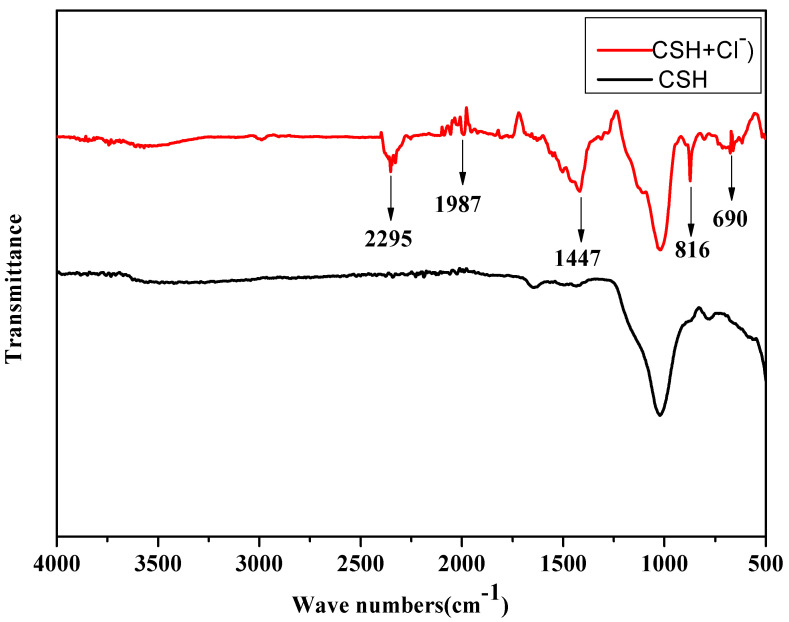
FTIR pattern comparison of CSH gel doped with chloride ions.

FTIR analyses were conducted to examine the modifications in composition and functional groups of CSH gels after chloride ions adsorption. The results are illustrated in Figure 7.

As can be seen from the figure, there was a characteristic absorption peak of ν(Si-O) Q^1^ in the vicinity of the wave number of 690 cm^−1^, and the absorption peak increased slightly after adsorbing chloride ions. This shows that the adsorption of chloride ion reduces the polymerization degree, the number of the bridging oxygen, and the chain length of siloxane tetrahedron in CSH gel, which is manifested by the increase of Q^1^ content in the infrared spectrum. There was a stretching vibration peak of -OH at the wave number of 3644 cm^−1^. According to the analysis, there was a certain degree of displacement between Cl^−^ and OH^-^, so the peak corresponding to -OH was weakened, indicating that CSH gel had adsorbed chloride ions.

#### 3.3.2. Structural Changes in CSH Gel

**Figure 8:**
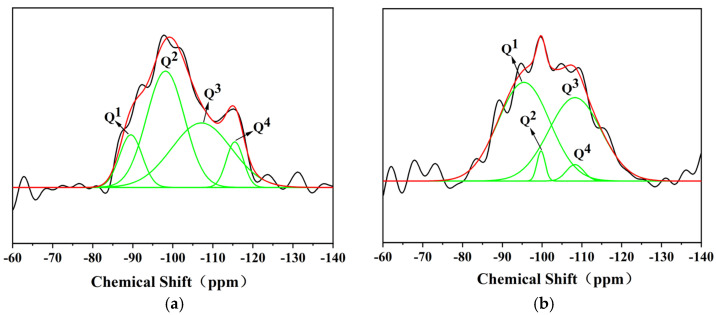
^29^Si NMR of CSH gel after adsorption of chloride ions. (**a**) CSH. (**b**) CSH + chloride ions. The black line is the test result, and the red line is the fitted curve.

As stated earlier, the composition and chemical bonding of the CSH gel were significantly affected by the adsorption of chloride ions. The effect of chloride ions on the structure of the CSH gel was analyzed using ^29^Si NMR spectroscopy, and the results are presented in Figure 8.

From Figure 8, it is evident that the polymerization states of the silicon-oxygen tetrahedra in the CSH gel structure prior to chloride ion adsorption were primarily represented by bimeric or chain-end groups (Q^1^ type), silicon-oxygen tetrahedra representing chain intermediate groups (Q^2^ type), silicon-oxygen tetrahedra corresponding to double-linked polymerization structures or layered structures (Q^3^ type), and silicon-oxygen tetrahedra with a three-dimensional network structure (Q^4^ type). After the adsorption of chloride ions, the peak shapes of these four types of polymerized silicon-oxygen tetrahedra exhibited minor changes and appeared as broad peaks, indicating a low crystallinity of the CSH gel.

Furthermore, following the adsorption of chloride ions, the peak area of Q^1^ in the CSH gel increased from 11.64% to 50.90%, while the peak area of Q^2^ decreased from 43.04% to 2.85%. This indicates that the relative content of Q^1^ increased while that of Q^2^ decreased, suggesting that chloride ions significantly affected the structure of the CSH gel and reduced its polymerization degree.

#### 3.3.3. Morphological Changes in CSH Gel

**Figure 9:**
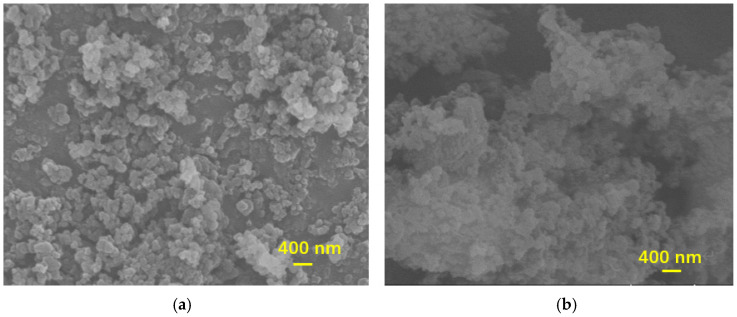
Morphology change in CSH gel after adsorption of chloride ions. (**a**) CSH. (**b**) CSH + chloride ions.

To investigate the impact of chloride ions on the morphology of CSH gels, scanning electron microscopy (SEM) was employed to observe the morphological changes in the CSH gels following ion adsorption, as illustrated in Figure 9.

From the figure, it can be observed that the morphology of CSH gels prior to chloride ion adsorption was predominantly irregular and spherical. However, after the adsorption of chloride ions, significant changes occurred in the CSH gel structure. A layer of chloride ions was adsorbed on the surface of the CSH gel, and the phenomenon of agglomeration became more pronounced. The primary reason for these morphological changes in the CSH gels following chloride ions adsorption was the replacement of hydroxide ions on the surface of the CSH gel by chloride ions. This replacement altered the structure of the silicon-oxygen tetrahedra, resulting in considerable changes in the morphology of the CSH gel.

**Figure 10:**
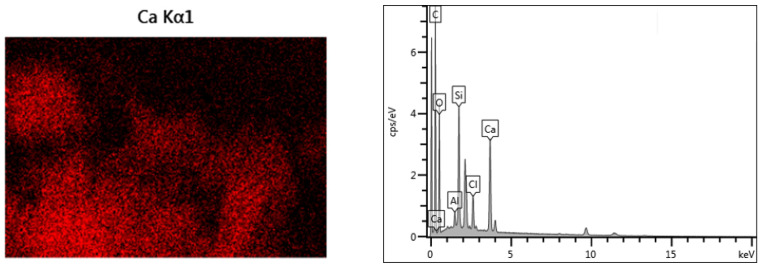
EDS of CSH gel after adsorption of chloride ions.

The elemental spectra and composition analyses of the CSH gel before and after chloride ions adsorption are presented in Figure 10 and Table 1. From the elemental energy spectrum and composition analysis, it is evident that after the adsorption of chloride ions, a notable amount of chloride ions was incorporated into the CSH gel. Specifically, the proportion of Cl detected in the spectroscopic analysis was found to be 4.44%. This indicates that CSH gel possesses a strong adsorption capacity for chloride ions.

**Figure 11:**
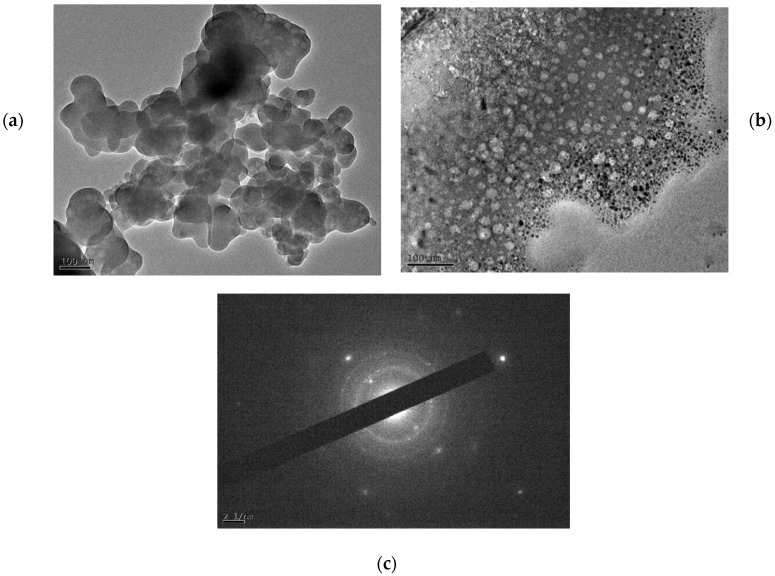
TEM observation and electron diffraction spectra of CSH gel. (**a**) CSH. (**b**) CSH + chloride ions. (**c**) Diffraction ring of CSH.

Considering electron diffraction and high-resolution electron microscopic analysis, the microscopic information of crystal morphology can be grasped more accurately. In this section, transmission electron microscopy was used to study the morphology of CSH gel before and after chloride ions adsorption. Transmission electron microscopy and electron diffraction patterns before and after chloride ions adsorption on CSH gel, as shown in Figure 11.

As shown in the figure, the CSH gel prior to chloride ions adsorption revealed a diameter of approximately 100 nm and exhibited a smooth crystal edge when observed under high-resolution transmission electron microscopy (HRTEM). In contrast, the electron diffraction patterns of the CSH gel after chloride ions adsorption exhibited significant differences. The morphology of the CSH gel after chloride ions adsorption appeared spherical, with a reduction in particle size and improved dispersion.

Additionally, the electron diffraction spectrum indicates that the diffraction pattern was centered around the transmission spot of the direct beam, with the presence of multi-crystalline concentric rings observed in the higher-order diffraction regions on the periphery. Furthermore, the intensity of electron diffraction spots varied in different directions, and there were differences in the brightness of the higher-order diffraction spots. This observation suggests that the crystal structure of CSH gel is complex.

These changes may be attributed to the chemical adsorption of chloride ions onto the CSH gel, leading to a displacement reaction between the adsorbed chloride ions and the hydroxide ion on the surface of the CSH gel, which ultimately alters the morphology of the CSH gel.

## 4. Conclusions

In this paper, we investigated the influencing factors and adsorption mechanisms of chloride ions on CSH gel. The adsorption of chloride ions in CSH gel is also influenced by the presence of cations in the solution. Specifically, when calcium ion is present, it promotes the growth of the average chain length of the CSH gel, thus enhancing its adsorption capacity for chloride ions. The adsorption of chloride ions in CSH gel primarily occurs through physical adsorption on the surface and, to some extent, within the internal pores of the CSH gel. Additionally, a displacement reaction occurs between hydroxide ions and chloride ions.

## Figures and Tables

**Table 1:** Component analysis of CSH gel after adsorption of chloride ions.

Element	Mass Percentage
C	48
O	33.5
Si	9.46
Ca	11.82
Cl	4.44

## Data Availability

The original contributions presented in the study are included in the article, further inquiries can be directed to the corresponding author.
